# Editorial: Psychopathological trajectories in adolescent and young adult psychiatry

**DOI:** 10.3389/fpsyt.2025.1517233

**Published:** 2025-02-18

**Authors:** Alejandro de la Torre-Luque, Idan Menashe, Eoin McElroy

**Affiliations:** ^1^ Department of Legal Medicine, Psychiatry and Pathology, Faculty of Medicine, Universidad Complutense de Madrid, Madrid, Spain; ^2^ Department of Public Health. Faculty of Health Sciences, Ben-Gurion University of the Negev, Be’er Sheva, Israel; ^3^ School of Psychology, Ulster University, Coleraine, United Kingdom

**Keywords:** adolescence, mental disorder, personalized medicine, lifelong approach, developmental psychopatholgy

Adolescence constitutes a crucial period in life, featured by critical influences of biological and maturational forces on physiological and psychological processes towards adulthood. People face important individual and social challenges when entering adulthood, such as the exploration of life directions and the entry into adult commitments. Alterations from normative patterns of adjustment are frequently seen during these ages, leading to psychiatric conditions that may show enduring effects later in life. In this regard, over one in four people may exhibit a psychiatric disorder during their adolescent and young ages (1, 2). Moreover, the development of a psychiatric condition in young ages (regardless of reaching a clinical meaningfulness point) can increase up to 12 times the risk of suffering from another condition later in life (3). In addition, the presence of a mental health disorder at a young age has been associated with an increased risk of mortality directly or indirectly, through the development of suicidal behaviour. Suicidal behaviour and non-suicidal self-harm (NSSH) are increasingly prevalent in adolescence according to both community and clinical research. Finally, the burden of disease and loss of functioning (i.e., years of life lost) derived from psychiatric disorders have increased by 35% in young people in recent decades (4).

From a lifelong standpoint, the study of mental health conditions in adolescence and youth becomes critical to understanding early psychopathology and psychiatric manifestations, and further episodes across the lifespan. Moreover, the identification of period-specific protective and risk profiles may help improve diagnostic tools and optimize treatment provision and follow-up protocols. Casey et al. (5) stressed the importance of focusing on critical periods of psychopathology development to study the natural course of psychiatric disorders across the lifespan, to identify potential developmental cascades in constant interaction with environmental influences. In this regard, the course of a neurodevelopmental process may depict different shapes with notable peaks across the life periods. These peaks may represent sensitive periods or restricted windows of development, featured by a greater susceptibility to external influences. A large body of evidence has put the spotlight on adolescence and youth as sensitive periods of psychopathology development.

This Research Topic entitled ‘*Psychopathological Trajectories in Adolescent and Young Adult Psychiatry*,’ comprises four outstanding studies aimed at providing robust evidence on psychiatric conditions and their interplay with protective and risk factors in adolescence and youth, understood as critical periods of psychopathology and NSSH development. The Research Topic presents a good variety of studies, obtaining evidence from qualitative and quantitative designs and from different regions across the globe. First, the study by Pace et al. constitutes a systematic review with meta-analysis on the use of an empirically based assessment system (ASEBA) to explore emotional symptoms and conduct problems in Italian adolescents. Overall, 44 studies were included in the analysis, comprising evidence from both cross-sectional and longitudinal studies. The analyses provided normative scores on emotional and conduct problems in adolescents, as well as some interesting effects of sociodemographic factors on emotional symptoms. For example, a higher proportion of female participants in the sample, higher age, and higher mean scores in studies published during the pandemic years were uncovered.

The second study by Xiao et al. constitutes an empirical, prospective study comprising a sample of 520 adolescents (hospital inpatients) with a psychiatric disorder and to describe the sociodemographic and clinical profiles of patients with self-harm behaviour (NSSH). The authors reported that more than 1 in four participants (77.8%) in their sample showed active NSSH. Furthermore, the factors associated with the group with NSSH were: female gender, having younger age (10–14 years), depressive symptoms, suicide history, and a history of adverse life events. Most of these findings are in-line with risk factors of NSSH that are commonly seen in studies across the globe, providing invaluable data from an eastern country with a large sample of adolescents in clinical settings.

From a wider, systemic perspective, Wang et al. collected data on parental experiences of parents from Chinese adolescent inpatients with an affective disorder and NSSH during the COVID-19 pandemic years. This study was based on a phenomenological approach to collect qualitative data. The study stressed several important points to incorporate within therapeutic interventions to reduce NSSH engagement, such as the knowledge gaps of parents in terms of dealing with NSSH crisis, management of expectations towards their sons and related role to maintain NSSH. Additionally, the study stresses the importance of parental processes to help adolescents resolve internal conflicts and external conflicting situations, and placing parental factors as critical risk factors for NSSH engagement.

Finally, the study by Lockwood et al. aimed to study the dynamics of NSSH in a sample of young people and adults who engaged in a NSSH episode repeatedly. The participants were asked to report about their experiences of self-harming by using the Card Sort Task for Self-harm (CaTS). Changes from the first NSSH episode to the most recent one were assessed. As a result, the authors stressed similarities and differences between the age groups in terms of NSSH dynamics. Additionally, some valuable insight on emotional regulation dynamics were obtained from this study. For instance, young people showed an attenuation in positive emotion states after self-harming from the first NSSH episode in comparison to a more recent one; in contrast, adult people who engaged in NSSH kept feeling better after self-harming. Moreover, adult participants showed higher negative emotional states the day after self-harming in comparison to their young counterparts.

In summary, this Research Topic constitutes an invaluable source of evidence, contributing to the advancement of personalized medicine of psychiatric disorders and gaining insight into a comprehensive understanding of clinical profiles in adolescence and youth, as critical periods of development with pivotal influence in adult and older life.
